# Predictors of Survival Outcomes After Primary Treatment of Epithelial Ovarian Cancer in Lagos, Nigeria

**DOI:** 10.1200/GO.20.00450

**Published:** 2021-01-15

**Authors:** Kehinde Sharafadeen Okunade, Adebola A. Adejimi, Ephraim O. Ohazurike, Omolola Salako, Benedetto Osunwusi, Muisi A. Adenekan, Aloy O. Ugwu, Adaiah Soibi-Harry, Olayemi Dawodu, Adeyemi A. Okunowo, Rose I. Anorlu, Jonathan S. Berek

**Affiliations:** ^1^ Department of Obstetrics & Gynaecology, College of Medicine, University of Lagos, Lagos, Nigeria; ^2^ Department of Community Health and Primary Care, College of Medicine, University of Lagos, Surulere, Lagos, Nigeria; ^3^ Department of Obstetrics & Gynaecology, Lagos University Teaching Hospital, Lagos, Nigeria; ^4^ Department of Clinical and Radiation Oncology, Lagos State University Teaching Hospital, Lagos, Nigeria; ^5^ Department of Anatomic and Molecular Pathology, College of Medicine, University of Lagos, Lagos, Nigeria; ^6^ Department of Obstetrics & Gynaecology, Stanford University School of Medicine, Stanford, CA

## Abstract

**PURPOSE:**

This study was designed to investigate the clinicopathologic predictors of progression-free survival (PFS) and overall survival (OS) in patients with epithelial ovarian cancer (EOC) following primary treatment in Lagos, Nigeria.

**MATERIALS AND METHODS:**

Using data from a retrospective cohort of 126 patients who received treatment for EOC between 2010 and 2018, we identified 83 patients with a complete clinical record for subsequent data analysis. Patients' demographics and updated 2-year follow-up status were abstracted from medical records. Kaplan-Meier survival curves were compared using the log-rank test, and Cox proportional hazard models were used for multivariate analysis to identify independent predictors of survivals following treatment in EOC patients.

**RESULTS:**

The median PFS and OS were 12 and 24 months, respectively. After adjusting for covariates in the multivariate analysis, younger age ≤ 55 years (hazard ratio [HR] = 0.40; 95% CI, 0.22 to 0.74; *P* = .01) and International Federation of Gynecology and Obstetrics (FIGO) stage I/II (HR = 0.02; 95% CI, 0.01 to 0.08; *P* = .01) were independent predictors of improved PFS, whereas being premenopausal (HR = 2.34; 95% CI, 1.16 to 4.75; *P* = .02) was an independent predictor of reduced OS after 2-year follow-up.

**CONCLUSION:**

PFS could be predicted by the age and FIGO stage of the disease, whereas menopausal status was predictive of OS in patients with EOC. This knowledge should form the basis for counseling patients with ovarian cancer during their primary treatment and lend support to the importance of aggressive follow-up and monitoring for the older, premenopausal patients and those with an advanced stage of epithelial ovarian cancer. However, robust longitudinal research should be carried out to provide additional reliable insight to this information.

## INTRODUCTION

Ovarian cancer (OC) is the eighth most common cancer among women worldwide and the eighth leading cause of cancer mortality. In 2018, there were almost 300,000 new patient cases and 184,799 deaths because of ovarian cancer.^1^ In Nigeria, it is the second most common gynecological cancer with an incidence of 30.5%.^2^ It is typically present in postmenopausal women with the peak incidence occurring in the early 60s.^3^ However, OC may also be seen in younger women, in which case it is often associated with certain genetic predispositions such as *BRCA1* and *BRCA2* gene mutations.^4^

**CONTEXT**

**Key Objective**
Are there clinicopathologic factors that predict progression-free survival (PFS) and overall survival (OS) in patients with epithelial ovarian cancer (EOC) following their primary treatment?
**Knowledge Generated**
The results indicated that following complete treatment of EOC with surgery and chemotherapy, patients ≤ 55 years of age and early International Federation of Gynecology and Obstetrics stage of the disease were the independent predictors of improved PFS, whereas being premenopausal was recorded as an independent predictor of poor OS after a 2-year follow-up.
**Relevance**
The findings from this study should form the basis for counseling patients with ovarian cancer during their primary treatment and lend support to the importance of aggressive follow-up and monitoring of the older premenopausal patients and those with an advanced stage of EOC.

Epithelial ovarian cancer (EOC) accounts for 90% of all histological types of OC^5,6^ with more than 70% of patients being diagnosed at the advanced stage.^3^ As a result of the asymptomatic nature and insidious onset of the disease, most of the cases are detected at an advanced stage.^7^ The standard first-line treatment at this advanced stage of the disease is optimal debulking surgery followed by adjuvant platinum-based chemotherapy^4^; or more recently, in women who are unfit for initial primary surgery or those in whom optimal primary debulking surgery cannot be achieved, the first-line treatment is neoadjuvant chemotherapy followed by interval debulking surgery.^4,8^ However, despite initial treatment and response, the recurrence rate at these advanced stages of the disease (International Federation of Gynecology and Obstetrics [FIGO], stages III-IV) may be as high as 80% usually because of chemotherapy resistance.^4^ There are two histological subtypes of EOC, and these include type I and II carcinomas.^9^ Type I carcinomas are generally slow-growing indolent neoplasms that have their precursor lesions in the ovaries,^10^ and these are endometrioid carcinoma, clear cell carcinoma, mucinous carcinoma, and low-grade serous carcinoma (LGSC). Type II or high-grade serous carcinomas (HGSC) are clinically aggressive neoplasms and may develop de novo from the tubal and/or ovarian surface epithelium. HGSC account for 68% of ovarian cancer and have the worst prognosis as a result of their aggressive pathologic features and usually being diagnosed at an advanced stage of the disease.^9^

To provide better information and personalized care to affected women, it is extremely valuable to identify the important predictors of outcome in patients with EOC in sub-Saharan Africa (SSA). Knowledge of these predictors will help to assess the efficacy of standard treatment and also the usefulness of planning and implementation of follow-up care.^11^ However, there is currently conflicting evidence on reliable independent predictors of survivor outcomes among patients with EOC following their complete primary treatment.^12-14^ A previous combined exploratory analysis of three prospectively randomized phase III multicenter trials that examined the role of surgical outcome as a prognostic factor in advanced EOC^15^ showed that complete tumor resection or optimal debulking is a predictor of improved progression-free survival (PFS) and overall survival (OS), whereas factors such as age, performance status, grade, FIGO stage, and histology are independent prognostic factors for OS. However, no similar study has been conducted among Black African women with EOC. This preliminary study was focused on identifying the clinicopathologic risk predictors of recurrence and death from EOC within 2 years after the primary treatment of affected women in Lagos, Nigeria. This will add to the available literature that contains studies predominantly conducted among mostly White participants in western countries of the world.

## MATERIALS AND METHODS

### Study Design

This was a retrospective cohort study that involved the review of case records of women with histologically confirmed EOC managed at the Lagos University Teaching Hospital (LUTH), Lagos, Nigeria, between January 2010 and December 2018.

### Eligibility Criteria

In this study, we included all patients who had a complete clinical record and relevant data for analysis and excluded patients with non-EOC and those who failed to commence treatment within 6 weeks of their presentation in LUTH. Data abstracted from patient medical records included age, parity, menopausal status, body mass index (BMI), serum cancer antigen (CA)-125 levels, coexisting morbidity (such as hypertension, diabetes mellitus, and cardiac, kidney, and liver diseases), type of primary treatment, surgical debulking status,^16^ presence of ascites, FIGO stage, histological subtype,^9^ PFS when historical information was available, and OS.

### Study End Points

The study end points were to determine the clinical and pathological characteristics that predict PFS and OS in patients with EOC. PFS was determined by calculating the interval from the time of completion of primary treatment to the first evidence of progression as determined by clinical examination, elevated tumor markers (serum CA125 and/or carcinoembryonic antigen), and/or radiological studies. OS was defined as the interval from the time of completion of primary treatment until death from all causes or last follow-up since completion of treatment for patients who were still alive. The survival data were censored after a 2-year follow-up.

### Statistical Analysis

Data analysis was performed using SPSS version 23.0 statistical package for Windows (IBM, Armonk, NY), and descriptive statistics were computed for all patient baseline characteristics. Characteristics of patients were described using mean and standard deviation (if normally distributed) or median and interquartile range (if skewed) for continuous variables and by frequencies and percentages for categorical variables. Kaplan-Meier estimates of PFS and OS time stratified by the various predictive factor categories were calculated and compared by employing the log-rank test statistics.^17^ Patients who were alive at the last follow-up or those without recurrence were censored. Multivariate Cox proportional hazard models were used to assess the association between participants’ clinicopathologic characteristics and survival outcomes while adjusting for other covariates. Backward stepwise conditional techniques were used to build the final multivariate models to include age and other variables using *P* < .2 to remain in the model. Associations are regarded as significant if *P* < .05. All *P* values are two-sided.

### Ethical Considerations

Ethical approval for this study was obtained from the Health Research Ethics committee of the LUTH (ADM/DCST/HREC/1912) before the review of case records and data collection. Ethical principles according to the Helsinki declaration were considered during this study.

## RESULTS

We recorded 126 patient cases of ovarian cancer managed in the hospital during the period under review, in which 83 were eligible for inclusion in the final analyses. Excluded from the analyses were 17 women with non-EOC, 11 who failed to undergo complete primary treatment or were lost to follow-up, three who did not commence treatment within 6 weeks of their cancer diagnosis, and 12 with insufficient clinical data.

The mean age of the patients in the study group was 54.4 ± 11.5 years. Patients were predominantly in the age group 50-59 years (n = 28, 33.7%), multiparous (n = 53, 63.8%), postmenopausal (n = 44, 53.0%), and with normal body weight (n = 37, 44.6%). A larger proportion of patients had primary debulking surgery as their first upfront treatment (n = 47, 56.6%) with the majority having FIGO stage III and IV diseases (n = 61, 73.5%) and high-grade serous carcinomas (n = 53, 63.9%). Sixty-three of the patients (75.9%) had documented tumor relapse, whereas 29 (34.9%) were reported to have died at the 2-year follow-up review in this study. The median PFS and OS at 2 years were 12 months (interquartile range, 6-24 months) and 24 months (interquartile range, 23-24 months), respectively (Table 1). The distribution of patients by recurrence and death at 2-years of follow-up is shown in Table 2.

**TABLE 1:**
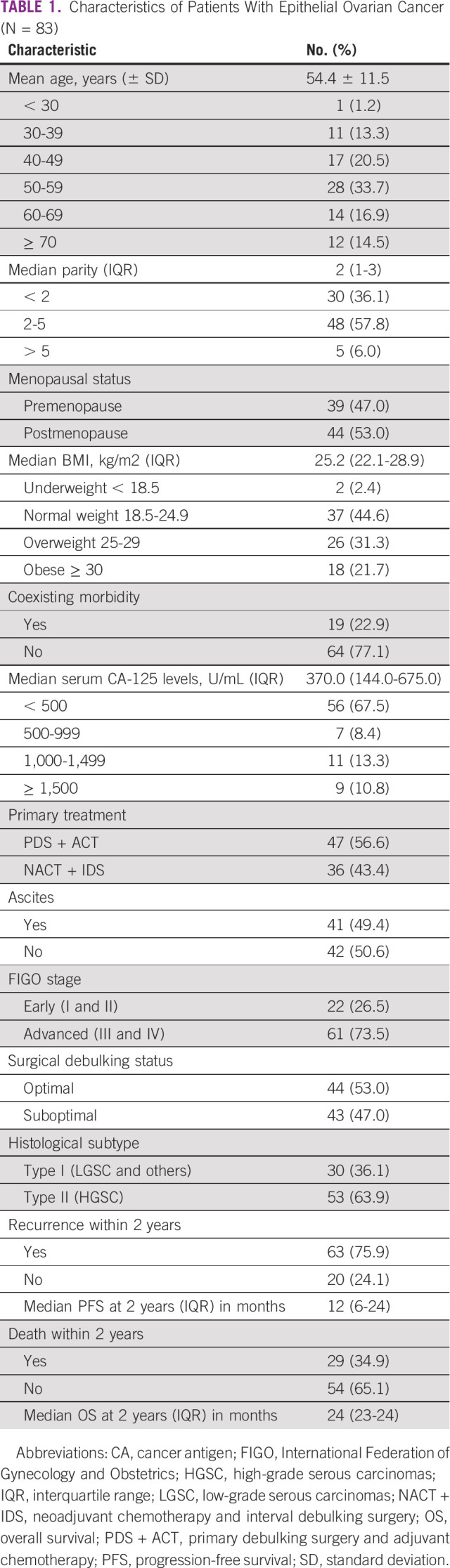
Characteristics of Patients With Epithelial Ovarian Cancer (N = 83)

**FIG 1:**
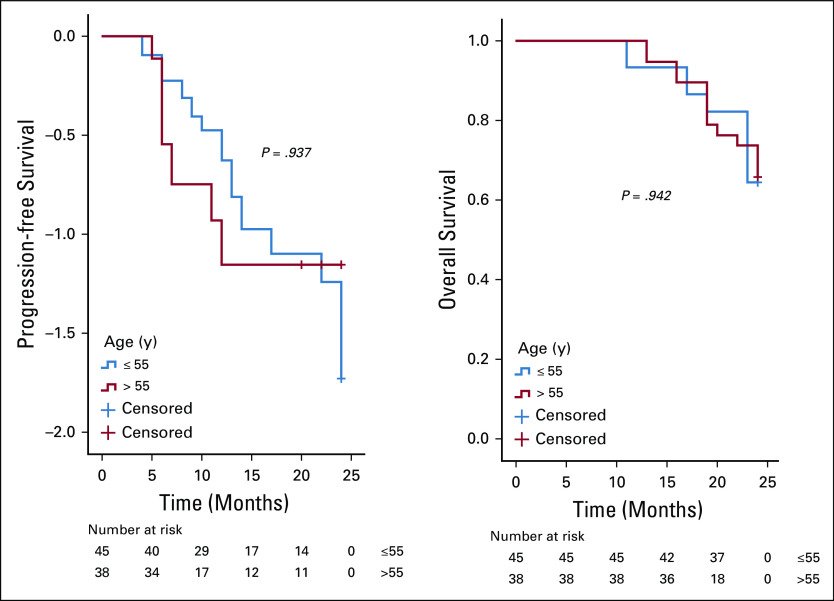
Kaplan-Meier curve of progression-free survival (PFS) and overall survival (OS) stratified by age—age (≤ 55 *v* > 55 years) was not associated with PFS (*P* = .937) and OS (*P* = .942).

**FIG 2:**
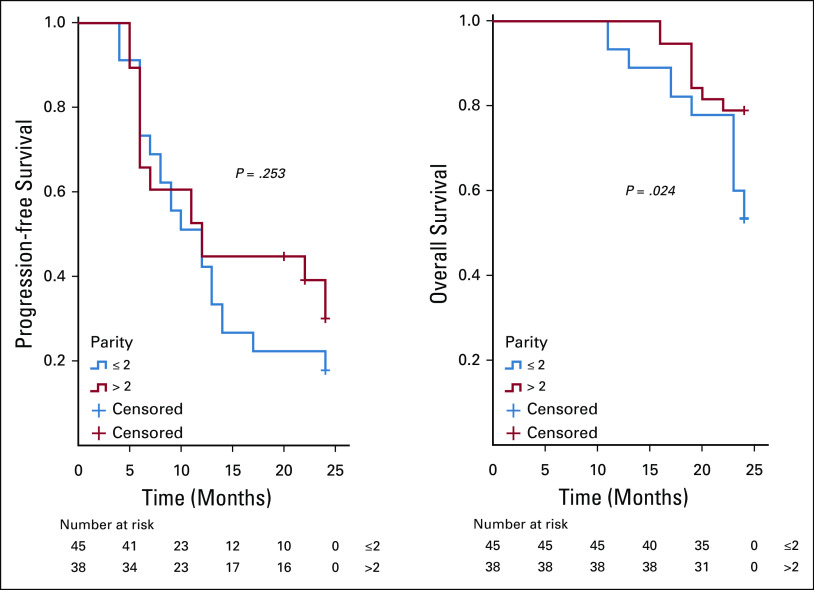
Kaplan-Meier curve of progression-free survival (PFS) and overall survival (OS) stratified by parity—the patient’s parity (≤ 2 *v* > 2) was associated with OS (*P* = .024) but not PFS (*P* = .253).

**FIG 3:**
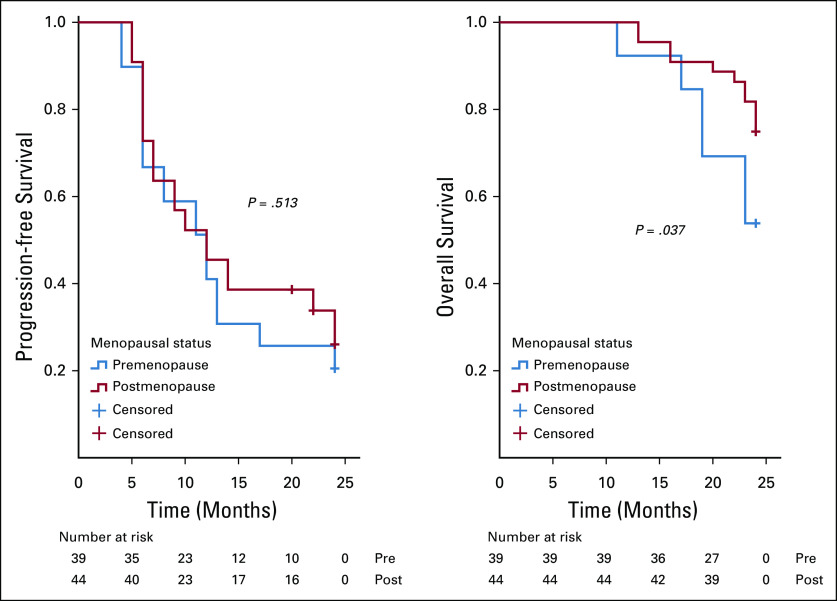
Kaplan-Meier curve of progression-free survival (PFS) and overall survival (OS) stratified by menopausal status—menopausal status (premenopause *v* postmenopause) was associated with OS (*P* = .037) but not PFS (*P* = .513).

**FIG 4:**
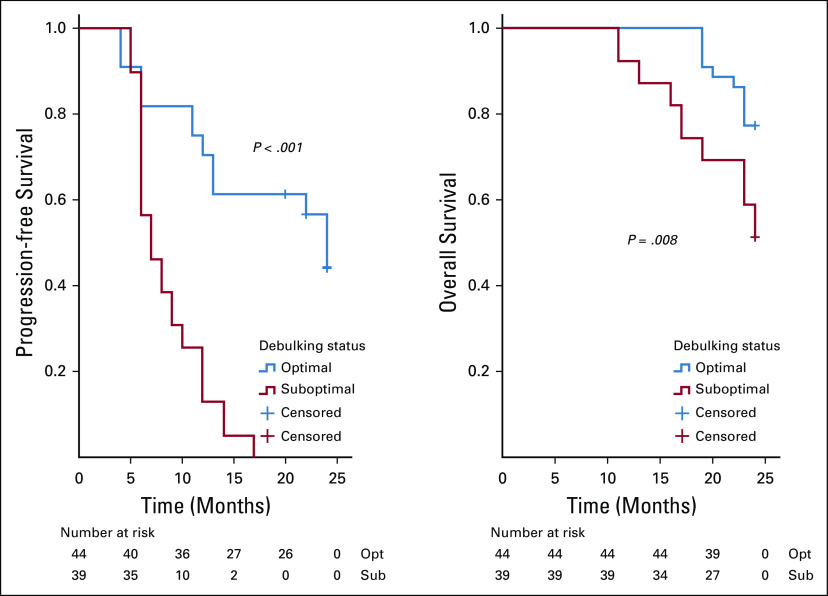
Kaplan-Meier curve of progression-free survival (PFS) and overall survival (OS) stratified by surgical debulking status—surgical debulking status (optimal *v* suboptimal) was associated with PFS (*P* = .001) and OS (*P* = .008). Optimal debulking is defined as when the residual disease is < 1 cm.^15^

**FIG 5:**
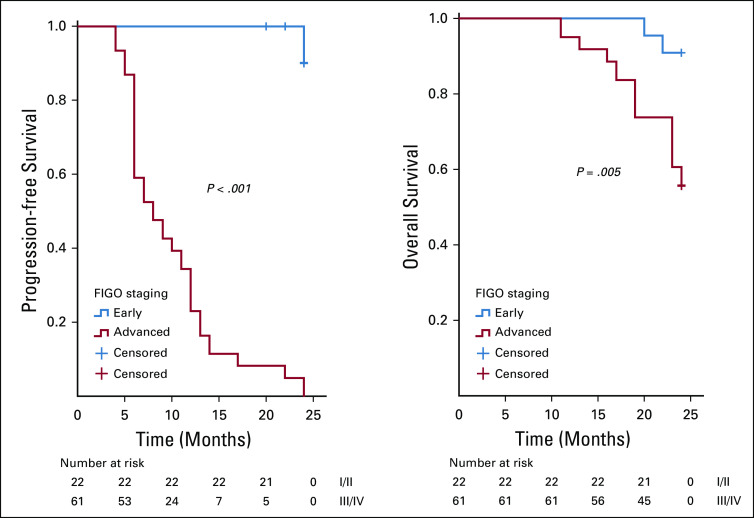
Kaplan-Meier curve of progression-free survival (PFS) and overall survival (OS) stratified by International Federation of Gynecology and Obstetrics (FIGO) staging—FIGO stage (early *v* advanced) was associated with PFS (*P* = .001) and OS (*P* = .005).

On analysis of survivals using the Kaplan-Meier estimates and log-rank statistics (Figs 1-5), it was found that age was not associated with PFS and OS, whereas both surgical debulking status and FIGO stage of the tumor were associated with survival outcomes. Parity and menopausal status were associated with OS only. After adjusting for covariates in the multivariate analysis, age ≤ 55 years (hazard ratio [HR] = 0.40; 95% CI, 0.22 to 0.74; *P* = .01) and FIGO stage I/II (HR = 0.02; 95% CI, 0.01 to 0.08; *P* = .01) were the only independent predictors of improved PFS (Table 3), whereas being premenopausal (HR = 2.34; 95% CI, 1.16 to 4.75; *P* = .02) was the only independent predictor of reduced OS at a 2-year follow-up (Table 4).

**TABLE 2:**
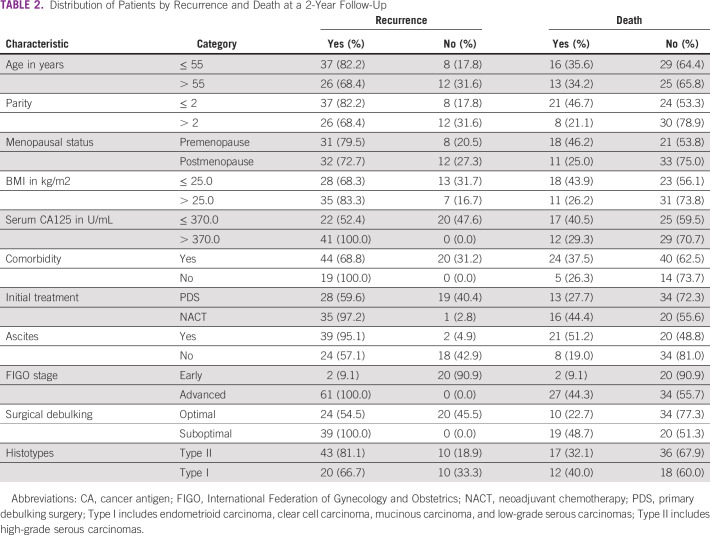
Distribution of Patients by Recurrence and Death at a 2-Year Follow-Up

**TABLE 3:**
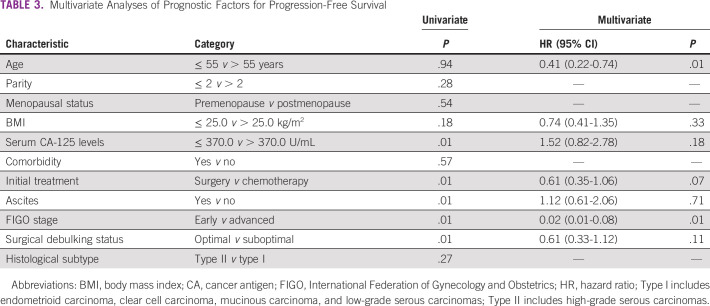
Multivariate Analyses of Prognostic Factors for Progression-Free Survival

## DISCUSSION

This study investigated the clinicopathologic predictors of survival outcomes in patients with EOC following primary treatment in Lagos, Nigeria. We found that 63 of the patients (75.9%) had documented tumor relapse, whereas 29 (34.9%) were not alive at completion of follow-up in this study. The patient’s age and FIGO stage predicted PFS, whereas menopausal status predicted OS.

**TABLE 4:**
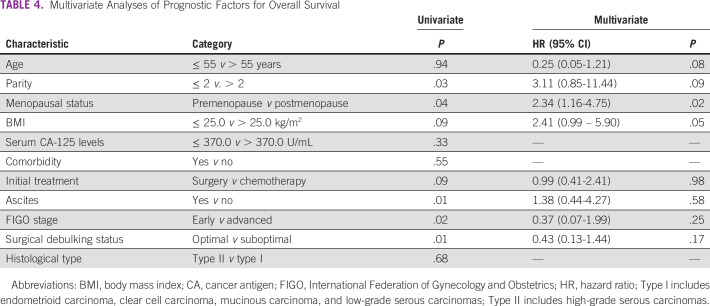
Multivariate Analyses of Prognostic Factors for Overall Survival

The rate of tumor recurrence recorded in this study (76.4%) is almost similar to the rate recorded in the report from a previous study conducted in the same setting in Lagos,^18^ whereas the high proportion of deaths recorded may be a reflection of the significantly large proportion of women (73.5%) who presented with an advanced stage disease together with its attendant poor survival outcome. Various studies outside SSA have focused on the identification of prognostic parameters for EOC, and several parameters that have been suggested to be predictive of survival in ovarian cancer include age,^19^ FIGO stage,^20^ postoperative residual tumor,^11,21,22^ tumor histology,^23^ histological grade,^24^ presence of ascites,^25^ and pretreatment serum concentrations of CA-125.^26^ An analysis of four prospective phase III intergroup trials in Germany that was conducted in 2016 found that patients less than age 40 years had a better PFS and OS compared with those older than 40 years.^27^ This is similar to the studies by Winter et al in 2007^19^ and Chang et al in 2015^28^ but in sharp contrast to the report from the study by Gil-Ibáñez et al^29^ where age was not found to be a predictor of survival in patients with ovarian cancer. Our current study showed a significant predictive effect of age on progression-free survival but not OS. This may be because age has an impact on patient ability to cope with stress related to a chronic disease state, and the altered physiology of the elderly alters the pharmacokinetics and pharmacodynamics of upfront chemotherapeutic agents used in the treatment of ovarian cancer.

According to the Gynecologic Oncology Group, an optimal surgical debulking is defined as when the residual disease is < 1 cm.^16^ Previous studies have shown that the ability to achieve optimal surgical debulking is the most important predictor of ovarian cancer survival,^30-36^ but this was not corroborated by our study. This may be due to the significant proportion of patients in this cohort (73.5%) who presented with advanced disease and because of the decreased likelihood of downgrading extensive disease by radical surgery and the poorer survival outcome of patients with high peritoneal cancer index, even after undergoing complete cytoreduction.^37^ We also reported that advanced FIGO stage of EOC independently predicted a reduced PFS similar to the finding by Yan et al^38^ and Liu et al^30^ but at variance with the report by Gil-Ibáñez et al.^29^ In contrast to our study, Liu et al^30^ reported in a study conducted in Tianjin, China, in 2014 that the patient’s tumor histotype is an independent prognostic factor for PFS in patients with EOC.

In contrast to our current study, Kim et al^39^ reported that a history of a previous parous event was associated with a significantly decreased mortality risk compared to nulliparity. This is not surprising as most parous women are older and often seek medical attention earlier compared with the younger nulliparous patients. Menopausal status is a well-known risk factor for ovarian cancer, and more than half of the patients diagnosed with ovarian cancer in our setting are postmenopausal^3^ as we reported in this study. There were few data regarding ovarian cancer survival in premenopausal patients; however, our study showed a reduced PFS in premenopausal patients unlike a previous study conducted by Trifanescu et al^36^ in 2018 where premenopausal patients with OC were shown to have a better oncologic long-term outcome. The finding of our study may be due to the relatively younger age of the premenopausal women in this cohort and the high prevalence of ovarian cancer subtypes (LGSC) associated with younger age at diagnosis that is relatively chemoresistant with its attendant high risk of relapse.^40^

Obesity is regarded as a major threat by increasing the incidence and mortality of different types of cancers.^41^ The problem is even more complex now considering the increasing incidence of obesity in most developing countries, including Nigeria, as a result of the rapid adoption of westernized lifestyles. However, at variance with data reported in the literature where increased BMI is a predictor of worst prognosis in patients with ovarian cancer,^35^ our study failed to show any relationship between BMI and ovarian cancer survival. In several epidemiologic studies that examined the association between serum CA-125 at diagnosis and survival from OC,^42-44^ there was an emphasis on the unique ability of CA-125 to independently predict OS in the setting of surgically defined disease status after primary therapy; however, our study did not find any strong association between CA-125 at diagnosis and survival in patients with EOC. This was despite our adoption of > 370 IU/mL as the cutoff in this study using stratification on the basis of the median levels of serum CA-125 recorded.

The major limitations of this study were its retrospective design that depended on effective documentation of patient history with the potential for missing data and the poor record-keeping system in our center, which resulted in the unacceptably high number of EOC patient cases with insufficient clinical data with the resultant small sample size and limited statistical power. Furthermore, the 2-year follow-up adopted may be too short for this type of study, and this may account for the relatively low OS recorded. This is also a single-center study, and thus, the findings may not be generalized to other geographical locations in Nigeria. However, this was the first study that assessed survival among women with EOC in SSA, and therefore, the preliminary data generated will form the basis for developing variables for a prediction model that will be validated in a future robust longitudinal study.

In conclusion, it is of extreme importance to identify the prognostic factors in patients with ovarian cancer to enable us to choose the most appropriate treatment strategy and to identify the risk of progression and death at follow-up. In our study, the patient age, menopausal status, FIGO clinical stage, and BMI were the only independent prognostic factors reported. The findings from this study further reiterate that once women are diagnosed with ovarian cancer, their age, the clinical stage of their disease, and their nutritional status are most likely to affect their survival. Consequently, these findings lend credence to the need for more intensive follow-up and monitoring after primary treatment for older premenopausal patients and those with an advanced stage of EOC. This can be achieved by developing a survival algorithm for triaging affected women to different approaches for immediate and long-term monitoring after completion of their primary treatment. However, a robust multicenter longitudinal study among Black African women is still required to provide additional reliable insight to this information.
